# An Optimization-Based Motion Planner for Car-like Logistics Robots on Narrow Roads

**DOI:** 10.3390/s22228948

**Published:** 2022-11-18

**Authors:** Lingli Yu, Hanzhao Wu, Chongliang Liu, Hao Jiao

**Affiliations:** 1 School of Automation, Central South University, Changsha 410083, China; 2 Beijing Institute of Automation Equipment, Beijing 100074, China

**Keywords:** motion planning, car-like robots, Timed-Elastic-Band, narrow roads

## Abstract

Thanks to their strong maneuverability and high load capacity, car-like robots with non-holonomic constraints are often used in logistics to improve efficiency. However, it is difficult to plan a safe and smooth optimal path in real time on the restricted narrow roads of the logistics park. To solve this problem, an optimization-based motion planning method inspired by the Timed-Elastic-Band algorithm is proposed, called Narrow-Roads-Timed-Elastic-Band (NRTEB). Three optimization modules are added to the inner and outer workflow of the Timed-Elastic-Band framework. The simulation results show that the proposed method achieves safe reversing planning on narrow roads while the jerk of the trajectory is reduced by 72.11% compared to the original method. Real-world experiments reveal that the proposed method safely and smoothly avoids dynamic obstacles in real time when navigating forward and backward. The motion planner provides a safer and smoother trajectory for car-like robots on narrow roads in real time, which greatly enhances the safety, robustness and reliability of the Timed-Elastic-Band planner in logistics parks.

## 1. Introduction

With the continuous development of service robot technology, the logistics industry is gradually transforming and upgrading. The traditional manual handling and logistics management model is being eliminated. The automation mode with logistics robots is widely used at the core of factories and production enterprises [1,2]. The intelligent operation of logistics handling has become the trend of industry development. Among them, motion planning is the key technology of autonomous navigation, which determines whether the robot can safely and efficiently reach the target. Motion planning is the search for a collision-free optimal trajectory from the current position to a local target in the dynamic environment around the robot [3]. Assuming that the localization [4], mapping [5], perception [6], and routing [7] modules perform well while the robot navigates on the wide-structured roads, this type of general motion planning approach has been well studied [8,9,10,11]. However, in logistics parks, robots travel on diverse and complex roads. Due to the size limitation of the park, the environment is mostly unstructured roads and presents a network or ring shape [12]. Unlike structured roads, unstructured roads generally do not have lane lines. Therefore, the sensors cannot recognize lane information. The drivable area cannot be determined based on the lane lines [13]. A safe path can only be reasonably planned based on the location of obstacles. To make matters worse, the park inevitably contains some narrow curves and straights. Due to the narrow exploration space, higher demands are placed on the robot motion planning capability. First, the robot needs to track the global path smoothly. To ensure the integrity of the goods, the robot’s speed during transport needs to be as smooth as possible. Second, the transportation process has the potential to involve reversing conditions. Non-holonomic constrained car-like robots also present a challenge in reversing on narrow roads. Finally, the robot often needs to interact with dynamic obstacles such as pedestrians and vehicles in the park during navigation. Ensuring collision-free transportation is the basis for realizing motion planning in the park. Various types of algorithms have been made available with poor applicability in this type of scenario [14]. Although the Timed-Elastic-Band algorithm is widely used in various mobile robot navigation systems for its advantages such as rapid solution and high applicability, it is still not sufficient to fully cope with these problems. Based on the above motivation, this paper proposes the Narrow-Roads-Timed-Elastic-Band (NRTEB) method for narrow space planning. This aims to improve the safety, stability and robustness of car-like logistics robots for motion planning on narrow roads. The main contributions of this paper are:

(1) We reconstruct the inner and outer workflow of the Timed-Elastic-Band algorithm to improve its motion planning performance on narrow roads.

(2) The jerk is added to the objective function in the form of a soft constraint, which improves the smoothness of the speed profile without significantly increasing the computation time.

(3) A strategy is designed to enhance safety when navigating in reverse, thereby reducing the risk of collision with narrow-road edges.

(4) A sampling-based obstacle avoidance strategy is proposed for the presence of obstacles on local targets to improve the safety and stability of narrow-road obstacle avoidance.

**Figure 1:**
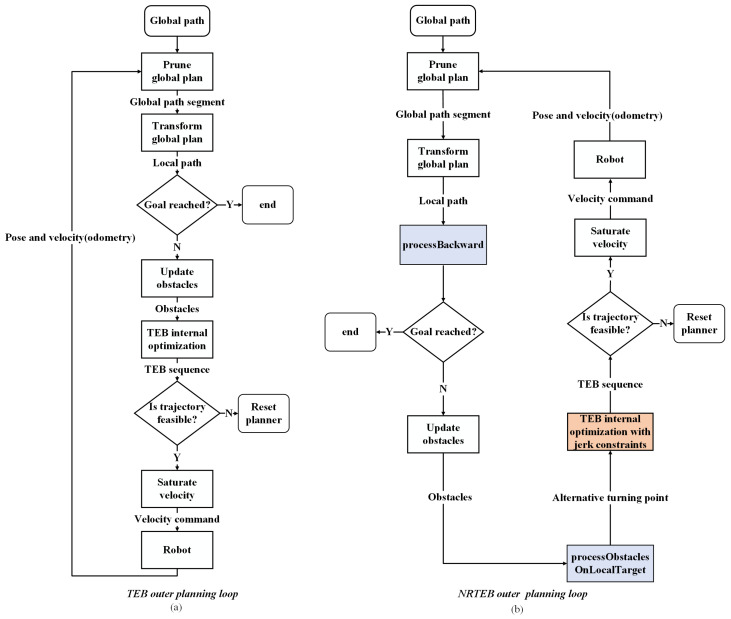
Workflow comparison. (**a**) is the workflow of the TEB method. (**b**) is the NRTEB workflow. The two modules in blue represent improvements in the outer planning loop, corresponding to contributions 3 and 4. The orange module represents improvements in the inner planning loop, corresponding to contribution 2.

A comparison of the method in this paper with the framework of the TEB is shown in Figure 1.

## 2. Literature Review

### 2.1. Researches on Point-to-Point Motion Planning Methods

For autonomous mobile robots that need to accomplish specific tasks, their operation process can be divided into segments of point-to-point motion planning [15]. For example, logistics robots continuously move from the current target to the next target in a set of freight handling processes. For point-to-point motion planning methods, they can be broadly classified into graph search-based ones, sampling-based ones, curve-interpolation-based ones, optimization-based ones and deep reinforcement learning-based ones [16]. One of the earliest point-to-point path planning methods is the graph search method. A well-known method is the shortest path algorithm proposed by Dijkstra [17]. In order to improve the search efficiency, the A* algorithm was proposed later [18]. This algorithm significantly reduces the search time by estimating the cost from the current location to the target through a heuristic function. The graph search algorithm is the most mature global path planning method, of which the A* algorithm has been widely used in the fields of autonomous driving and robotics. However, it only considers static obstacles and the path does not contain time information, so it is not applicable to dynamic local trajectory planning. Sampling-based schemes were initially studied to deal with pathfinding problems in high-dimensional spaces. One of the most representative is the rapid-exploration random tree (RRT) [19] method. A series of sampling-based studies were then enriched, such as RRT* [20] and informed-RRT* [21]. These both aim to improve the sampling efficiency and thus speed up the pathfinding process, but the paths are usually not smooth enough and optimality is not guaranteed. Curve interpolation methods are often used to generate smooth, comfortable trajectories. Common interpolated curves are polynomial [22], Bezier [23], and spline curves [24]. Since they are interpolation operations based on waypoints, the quality of local trajectory planning results depends on the quality of global path planning. In recent years, numerical optimization-based methods have attracted a great deal of attention [25]. This class of methods models the search problem as an optimization problem. Various constraints such as velocity, acceleration, minimum steering radius, etc. are incorporated into a unified model for solving the problem. Because of its ability to explicitly handle dynamic obstacles and various types of constraints, it is widely used in various autonomous driving and robotic systems. For example, Baidu’s motion planning module for unmanned vehicles extensively uses this type of approach to generate a short trajectory that is safe, comfortable, and collision-free [26]. In the field of robotics, the most popular one belonging to this category is the TEB local trajectory planner [27]. However, the TEB method ignores the comfort of speed change [28], which is detrimental to logistics handling robots. Although deep reinforcement learning-based motion planning methods are becoming a hot research topic, most of the work is still in the simulation stage. Such methods have not been adopted in the industry for the direct control of robots because safety and stability cannot be guaranteed [29].

### 2.2. Studies on Narrow Road Motion Planning Methods

A small amount of literature exists in the area of intelligent driving in narrow-road scenarios. The “narrow road assistant” proposed in [30] provided drivers with collision warning and steering support for driving on narrow roads. The driver-adapted ADAS proposed in [31] matched the driver’s driving style to provide appropriate steering assistance. Sato, T. et al. [32] presented two driving assistance system models based on drivers’ cognitive processes that are useful for achieving smooth passage on narrow roads and improving driver performance. In addition to advanced driver assistance systems applied on structured roads, some researchers have explored methods for the motion planning of mobile robots in narrow, unstructured road environments. Tian et al. [33] proposed a local path planning method for turning around in a narrow environment which combines Bezier curve and the minimum turning radius arc. The safety of planning in narrow roads is improved, but the real-time performance is poor. Li et al. [34] improved the planning performance of RRT in narrow roads or cluttered environments by fusing bidirectional RRT and rapidly exploring random vines. However, these paths are not smooth. Therefore, they cannot be provided to car-like robots for execution.

### 2.3. Studies on Timed-Elastic-Band Methods

In the scope of the TEB method, a variety of improved algorithms have successively been proposed. TEB adopts a strategy of predictive control to guide the robot to accomplish online trajectory planning tasks in a dynamic environment [35]. The first control action is finally adopted as the robot’s command [36]. The TEB planning algorithm proposed by C. Rösmann [37] is an evolution of the elastic band (EB) [38], which explicitly adds the time intervals to its optimization. The original TEB planner might cause a trajectory across obstacles which are owed to the optimization being trapped in local minima. Subsequently, C. Rösmann proposed an improved method for generating alternative suboptimal trajectory clusters based on distinctive topologies [39]. After the TEB algorithm was adopted by the ROS navigation stack [40], it has gradually become a hot topic of study among scholars. Keller et al. [41] studied the optimal obstacle avoidance trajectory generation method with the TEB framework. Their approach improves the obstacle avoidance capability of the TEB, but does not talk about the real-time planning problem. Ulbrich et al. [42] proposed a variant of TEB that uses loose ends in planning to produce more human-like paths. However, the authors admit that the method still has the probability of falling into local minima and failing to provide safe trajectories. S. Smith et al. [43] presented an egocentric map representation for TEB in an uncertain environment to solve the problem of mismatch between optimization graph and grid-based map. This reduces the planning time but is only validated in a simulated environment, so it is not sufficiently convincing to provide guidance for a real robot. A car-like sweeping robot system based on TEB local trajectory planning is proposed in [15]. The method improves the sweeping coverage, but the speed change during navigation is not smooth. The previous works have more or less improved the trajectory smoothness, solution stability, and obstacle avoidance safety of TEB. However, none of them can perfectly solve all the problems of TEB in narrow-road planning, especially the reverse planning and reverse obstacle avoidance scenarios that logistics robots often encounter. In addition, most of the studies only show the optimization effect of a certain aspect. There is a lack of real-time results presentation, which is very important for motion planning. Therefore, this paper intends to comprehensively address the problems arising from TEB in narrow-road planning. The effectiveness and superiority of the method is verified by simulation experiments and real-world experiments. This will provide good guidance for car-like robots driving on the narrow roads of logistics parks.

There are several reasons why the TEB algorithm can be widely used in robot motion planning: (1) Strong solving ability: To reduce costs, logistics robots usually have limited computational resources. TEB is able to solve optimization problems very quickly. In the objective function of TEB optimization, many error terms are partial [37]. As such, the Hessian matrix is sparse so that the solution is rapid. (2) Wide applicability: TEB is not only applicable to differential wheels but also the chassis of the Ackermann structure [44]. This makes a lot of sense for ROS [45] because, in the early stage of development, ROS studied mostly round universal wheel robots and rarely considered car-like robots [46]. In addition, TEB considered different vehicle chassis contour models, different obstacle types, and whether the obstacles are dynamic, so it is more adaptable to obstacle avoidance in dynamic environments. (3) Insensitive to the smoothness of global reference path: TEB selects the farthest point of the global path within the local sensing range as the local planning terminal. The shape of the trajectory is adjusted by various constraints, so it does not require the global path to conform to the kinematic constraints of the robot. Unlike Baidu Apollo’s local planner [26], it heavily relies on global path smoothing, so it reduces the overhead time of path smoothing. However, there are still the following problems that limit its application to car-like robots on narrow roads. (1) Speed planning results are not smooth: Although the non-holonomic constraint ensures smooth paths, both angular and linear speed profiles are not smooth. This is due to the fact that the TEB does not constrain the jerk of the entire trajectory. The unsmoothed speed planning results mean that there is a possibility of unstable control, which is contrary to the need for the robot motion and steering to be as smooth as possible in narrow-road scenarios. (2) Narrow road reversing planning is flawed: At the beginning of a new planning cycle, the TEB method selects a waypoint from the global path as a local target based on the forward-looking distance. When the global path is behind the robot, the heading of the local target is opposite the robot heading, because the global planning starts from the robot and points to the target. Based on this problem, TEB generates a parking trajectory, which is unacceptable for reversing behavior in confined environments. The robot is highly susceptible to collisions with road edges or cannot turn around due to the minimum turning radius. (3) Planning fails when obstacles fall on the local target: A local target is a waypoint obtained from the global path. Its position is fixed at the beginning of the planning cycle. If at this point the sensors detect a dynamic obstacle moving to the position of the local target, it will cause the planning to fail because the target is unreachable. This can be solved by updating the global path, however, global planning is unreliable as the module with the slowest update frequency and computation takes a long time. Additionally, the introduction of dynamic obstacles makes the search space non-convex and may lead the optimization into local minima.

## 3. Materials and Methods

For the above-mentioned problems arising in narrow road environments, we reconstruct the inner and outer workflow of the TEB algorithm. The improvements in the internal optimization loop are presented first, corresponding to the orange module in Figure 1. Then, the two working modules in the external working loop are introduced, corresponding to the blue modules in Figure 1.

### 3.1. Motion Smoothing

#### 3.1.1. Motion Planning Modeling

**Figure 2:**
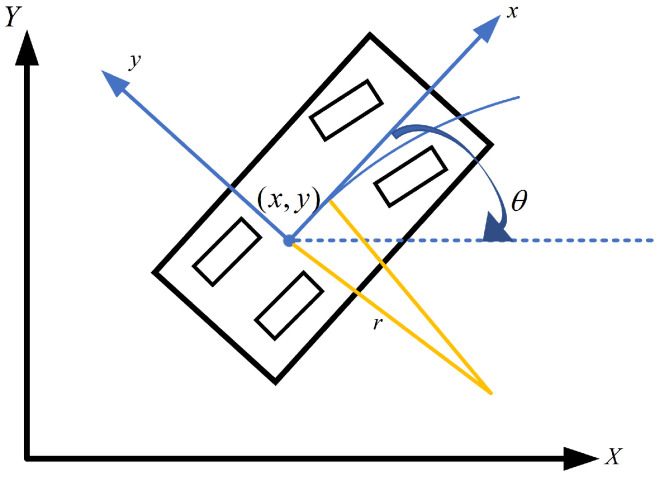
Robot kinematic model.

Car-like robots with an Ackermann structure traveling on the configuration space, which is shown in Figure 2. This paper adopts the kinematic model [47] for the Ackermann chassis.

(1) $$\left\{\begin{array}{c} \dot{x}=v\cos\theta \\ \dot{y}=v\sin\theta \\ \dot{\theta }=\frac{v\tan\delta_{f}}{L} \end{array}\right.$$

where (x,y,θ) is the configuration space state, (x,y) is a two-dimensional Cartesian coordinate, θ denotes the heading angle of the robot, *v* is the longitudinal speed, $\delta_{f}$ is the front steering angle, and *L* is the wheelbase of the vehicle.

The time intervals between poses are added to the optimization variables. Therefore, TEB not only modifies the geometry of the trajectory but also optimizes the velocity and acceleration, thus solving the high-dimensional search problem of motion planning. The sequence of poses in configuration space is expressed as:

(2) $$Q={\left\{q_{i}\right\}}_{i=0\ldots n-1}\;n\in N$$

where $q_{i}=\left(x_{i},y_{i},\theta_{i}\right)$. This denotes a robot pose in the configuration space.

The time interval between each two poses is denoted by ΔT, and the set of time interval vertices is expressed as:

(3) $$\tau ={\left\{\Delta T_{i}\right\}}_{i=0\ldots n-2}\;n\in N$$

where $\Delta T_{i}$ is the time interval between $q_{i}$ and $q_{i+1}$.

Thus, the TEB trajectory is represented as a combination of a sequence of poses and a sequence of time intervals, which are the optimization variables of the subsequent solution:

(4) S={Q,τ}

To facilitate the transformation of multi-objective optimization problems into graph optimization problems, the TEB algorithm transforms the hard constraint into a soft one. Therefore, this paper adopts the piecewise continuous, differentiable uniform cost function described in [27] to penalize the violation of constraints:

(5) $$e\left(x,x_{r},\epsilon ,R,n\right)\approx \left\{\begin{array}{cc} {\left(\frac{x-(x_{r}-\epsilon )}{R}\right)}^{n} & if\;x>x_{r}-\epsilon \\ 0 & otherwise \end{array}\right.$$

where $x_{r}$ presents the bound value. *n* is the polynomial order, *R* is the scaling, and ϵ expresses the safety margin. In this paper, we choose $x_{r}$ = 0.5, ϵ = 0.05, *R* = 0.25, *n* = 6.

The multi-objective function of TEB can be expressed as:

(6) $$f_{TEB}\left(S\right)=\sum_{k}\gamma_{k}\cdot f_{k}\left(S\right)$$

where $f_{k}\left(S\right)$ represents the deviation of each constraint from the desired value, and $\gamma_{k}$ denotes the corresponding weights. $f_{v}=e\left(v_{i},v_{max},\epsilon ,R,n\right)$ represents the velocity constraint of the trajectory, where $v_{i}$ is obtained by differentiating the adjacent pose $q_{i}$,$q_{i+1}$ from their time intervals $\Delta T_{i}$. $f_{a}=e\left(a_{i},a_{max},\epsilon ,R,n\right)$ represents the acceleration limitation of the trajectory, where $a_{i}$ is obtained from the three adjacent poses $q_{i},q_{i+1},q_{i+2}$ with their two time intervals $\Delta T_{i},\Delta T_{i+1}$. $f_{path}=e\left(q_{i},p_{i},\epsilon ,R,n\right)$ is the target-following constraint. $f_{obs}=e\left(-d_{min,j},-r_{min},\epsilon ,R,n\right)$ indicates that the obstacle constraint for those poses that are less than the minimum safe distance $r_{min}$. $f_{time}={\left(\sum_{i=1}^{n}\Delta T_{i}\right)}^{2}$ is the shortest time constraint. $f_{nh}$ represents a nonholonomic constraint between two adjacent poses which guarantees the generation of a trajectory that conforms to the vehicle kinematics. We refer to [39] for more details on constraint modeling.

**Figure 3:**
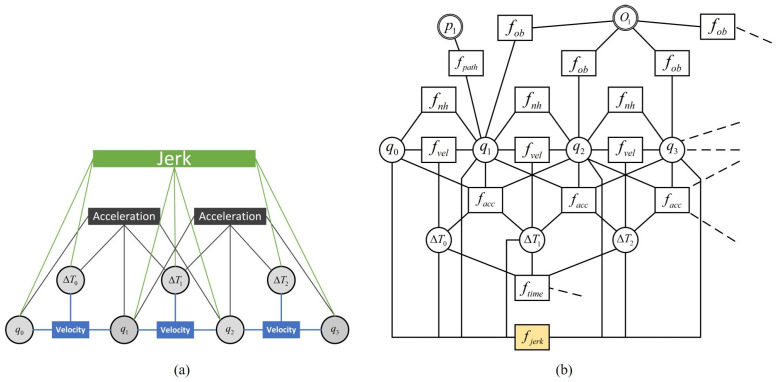
(**a**) A minimum hyper-graph considering jerk. (**b**) A simplified structure of the NRTEB hyper-graph. Circles represent optimized vertices and rectangles represent constrained hyperedges. Every seven adjacent vertices have a jerk super-edge, thus limiting the jerk of the whole trajectory.

Finally, Equation (6) is transformed into a graph optimization problem, as shown in Figure 3b. It is solved with the optimization framework of G2O [48] to obtain the optimal motion:

(7) $$S^{*}=\underset{S}{\arg\min}f_{TEB}\left(S\right)$$

where $S^{*}$ is the optimal TEB trajectory obtained from the optimal solution.

#### 3.1.2. Jerk Limitation

The linear jerk is the derivative of linear acceleration while the angular jerk is the derivative of angular acceleration. Constraining the jerk smooths out the speed profile of the trajectory, thus increasing the stability of the motion on narrow roads. A linear jerk is derived by the difference method from the four adjacent poses of *S* and the three time intervals between them, as shown in Figure 3a. The linear velocity is first represented by the sequence of poses *q*:

(8) $$\begin{array}{cc} v_{i} & =\frac{q_{i+1}-q_{i}}{\Delta T_{i}}\\ \; & =\frac{\sqrt{{\left(x_{i+1}-x_{i}\right)}^{2}+{\left(y_{i+1}-y_{i}\right)}^{2}}}{\Delta T_{i}} \end{array}$$

The linear acceleration is obtained from the three adjacent poses with their two time intervals, and thus the linear acceleration is represented by the linear velocity $v_{i},v_{i+1}$ and the time interval $\Delta T,\Delta T_{i+1}$:

(9) $${a_{v}}_{i}=\frac{2(v_{i+1}-v_{i})}{\Delta T_{i}+\Delta T_{i+1}}$$

Finally, the linear jerk is represented by the acceleration ${a_{v}}_{i},{a_{v}}_{i+1}$ with their three adjacent time intervals $\Delta T,\Delta T_{i+1},\Delta T_{i+2}$ as follows:

(10) $${J_{v}}_{i}=\frac{{a_{v}}_{i+1}-{a_{v}}_{i}}{\alpha \Delta T_{i}+\beta \Delta T_{i+1}+\gamma \Delta T_{i+2}}$$

In order to obtain the exact difference result, it is necessary to ensure that α+β+γ=1. $\Delta T_{i+1}$ is correlated with both ${a_{v}}_{i}$ and ${a_{v}}_{i+1}$, as seen from Figure 3. $\Delta T_{i}$ and $\Delta T_{i+2}$ are only correlated with ${a_{v}}_{i},{a_{v}}_{i+1}$, respectively. Thus, $\Delta T_{i+1}$ is given greater weight. This paper let α=γ=0.25,β=0.50.

Similarly, the angular jerk can be obtained as follows:

(11) $${J_{\omega }}_{i}=\frac{{a_{\omega }}_{i+1}-{a_{\omega }}_{i}}{\alpha \Delta T_{i}+\beta \Delta T_{i+1}+\gamma \Delta T_{i+2}}$$

where $a_{\omega }$ indicates the angular acceleration of the robot.

The constraint function of the linear jerk and angular jerk is expressed as:

(12) $$\begin{array}{c} f_{J_{v}}=e\left(J_{v_{i}},{J_{v}}_{max},\epsilon ,R,n\right)\\ f_{J_{\omega }}=e\left(J_{\omega_{i}},{J_{\omega }}_{max},\epsilon ,R,n\right) \end{array}$$

where ${J_{v}}_{max}$ is the threshold of the linear jerk and ${J_{\omega }}_{max}$ is the threshold of the angular jerk.

Therefore, the multi-objective function of NRTEB can be expressed as:

(13) $$f_{NRTEB}\left(S\right)=f_{TEB}\left(S\right)+\gamma_{J_{v}}f_{J_{v}}+\gamma_{J_{\omega }}f_{J_{\omega }}$$

where $\gamma_{J_{v}}$ is the optimization weight of the linear jerk constraint and $\gamma_{J_{\omega }}$ is the optimization weight of the angular jerk constraint.

With the above definition, the multi-objective problem is transformed into a graph optimization problem, where four poses and three time intervals constitute the minimum hyper-graph considering jerk, as shown in Figure 3a. A simplified example structure of the NRTEB hyper-graph is shown in Figure 3b.

### 3.2. Reverse Planning Enhancement

**Figure 4:**
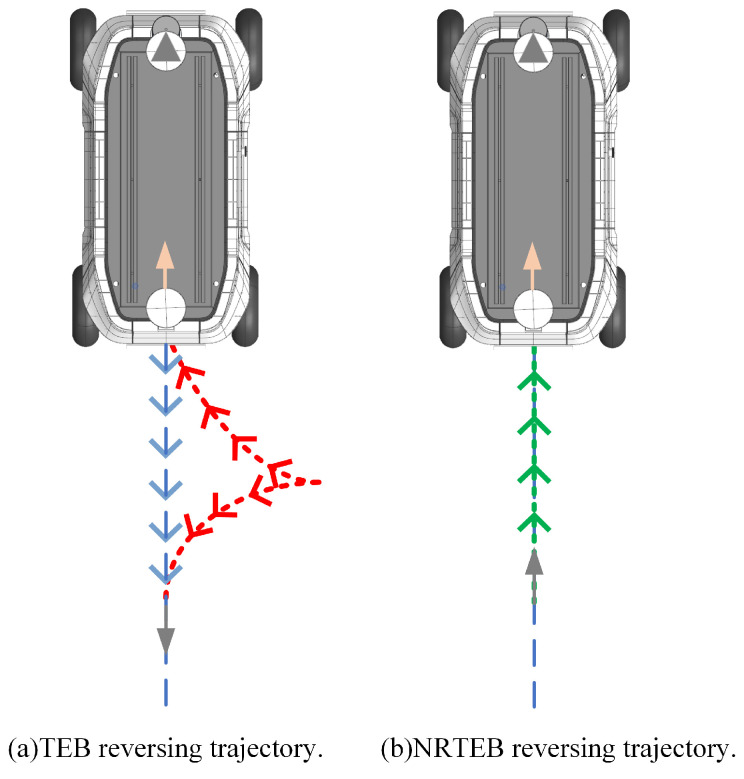
Reverse trajectory comparison. The blue dashed line indicates the global path. The red dashed line is the parking trajectory of the TEB. The green dashed line is the direct reversing trajectory of NRTEB. The arrow indicates the heading of the path point.

This paper has already explained the shortcomings of the original TEB in reverse planning in Section 2.3. Due to the problem of local target reference heading, TEB always tries to generate a parking trajectory when the global path is behind the vehicle, as shown in Figure 4a. This reversing strategy is extremely unsafe in narrow roads. To enhance the safety and robustness of reversing planning, this paper intends to solve the problem from local trajectory planning cycle instead of the global planning module. When the global path is behind the vehicle and the robot is far from the global target, NRTEB is able to generate a direct reversing trajectory, as shown in Figure 4b. When the robot approaches the global target, it takes this goal as the end point of optimization. As such, the robot’s terminating heading problem is also solved.

**Figure 5:**
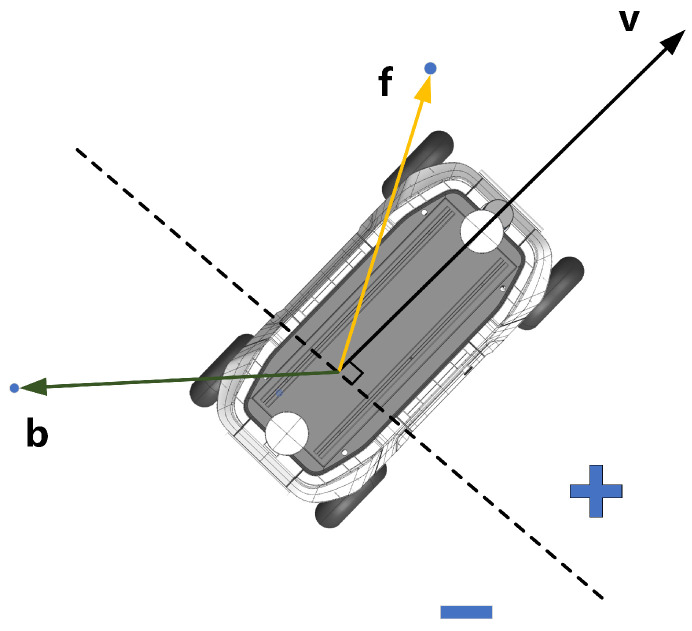
Relationship between the location of given points and the robot.

A vector product is used to represent the position of a point in relation to the robot. If the vector product of the body direction vector and vector formed by the selected point and the robot center of mass is negative, the picked point is considered to be behind the robot, i.e., Point *b* in Figure 5. The reference heading of the local target is corrected by judging this condition, so as to keep the heading of the planning start and the planning end consistent. The workflow for handling reversing is shown in Algorithm A1 in Appendix A, which indicates the corresponding processBackward module in Figure 1.

### 3.3. Obstacle Avoidance Strategy

**Figure 6:**
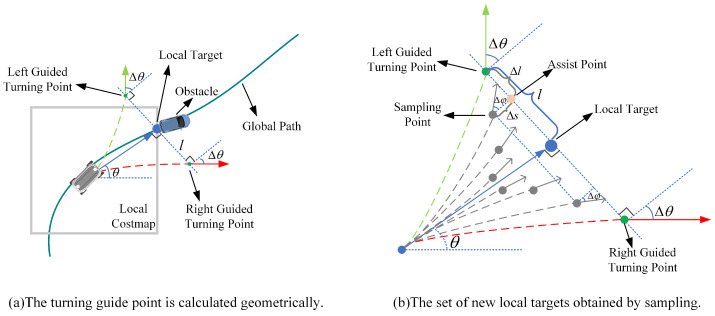
An obstacle avoidance strategy to cope with dynamic obstacles falling on local targets.

The essence of TEB planning is to periodically solve an optimization problem. The search space becomes non-convex when dynamic obstacles appear on the local targets, which leads to an optimization that may fall into local minima. In order to make the local target reachable and convexize the search space, an obstacle avoidance strategy that samples first and searches later to determine the local target location is proposed as shown in Figure 6.

Firstly, as shown in Figure 6a, the NRTEB planner calculates the direction vector from the robot to the local target $v_{dir}$, then make a vertical line of the direction vector across the local target, finds the location of the possible guided turning point on the vertical line based on the amount of lateral displacement change *l* and angle change Δθ. The green dashed line is the expected obstacle avoidance trajectory after updating the local target. The coordinates of the old local target are known: $\left(x_{lt},y_{lt}\right)$, the pose of the left guided turning point is obtained from the geometric relationship as:

(14) $$\left\{\begin{array}{c} x_{gtl}=x_{lt}-l\sin\theta \\ y_{gtl}=y_{lt}+l\cos\theta \\ \theta_{gtl}=\theta +\Delta \theta \end{array}\right.$$

where Δθ and *l* are the parameters determined by the working environment. From the symmetry relationship, the pose of the right-guided turning point is:

(15) $$\left\{\begin{array}{c} x_{gtr}=x_{lt}+l\sin\theta \\ y_{gtr}=y_{lt}-l\cos\theta \\ \theta_{gtr}=\theta -\Delta \theta \end{array}\right.$$

Secondly, on the basis of guided turning points, the set of obstacle avoidance points is sampled according to the preset parameters and geometric relationships, as shown in Figure 6b. Then, the set of sampling points is traversed until a point that meets the collision constraint is found as the new local target for the current planning cycle.

To obtain the sampling point, the assist point is first calculated according to Equation (16):

(16) $$\left\{\begin{array}{c} x_{as}=x_{gt}+\Delta l\cos\left(\frac{\pi }{2}-\theta \right)\\ y_{as}=y_{gt}-\Delta l\sin\left(\frac{\pi }{2}-\theta \right) \end{array}\right.$$

where $\left(x_{gt},y_{gt}\right)$ is the position of guided turning point and Δl denotes the determined lateral variation.

With the help of an assist point, the sampling point is then calculated by Equation (17):

(17) $$\left\{\begin{array}{c} x_{sp}=x_{as}-\Delta s\cos\theta \\ y_{sp}=y_{as}-\Delta s\sin\theta \\ \theta_{sp}=\theta +\Delta \theta -\Delta \phi \end{array}\right.$$

where $\left(x_{sp},y_{sp},\theta_{sp}\right)$ is the pose of sampling point, Δs denotes the determined longitudinal variation, and Δϕ denotes the determined angular variation. The decision process for the local target is shown in Algorithm A2 in Appendix A, which indicates the corresponding processObstacleOnLocalTarget function in Figure 1.

Finally, the algorithm introduces two hyperparameters, *l* and Δθ, which represent the amount of lateral distance and angle change, respectively. There is a relationship between the choice of these two parameters and the minimum turning radius because the car-like robot is subject to non-holonomic constraints. A simple proof is as follows:

Assume that the distance between the robot and the local target is *s*, according to the Pythagorean theorem:

(18) $$e=\sqrt{l^{2}+s^{2}}$$

**Figure 7:**
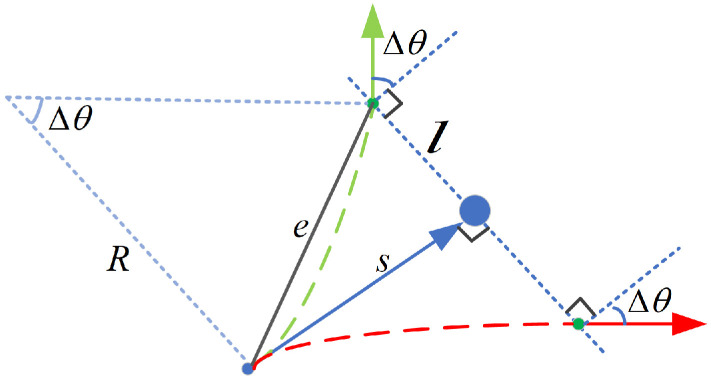
The geometric relationship between the obstacle avoidance parameters and the robot turning radius.

The robot kinematics are geometrically related as shown in Figure 7, so the relationship between Δθ and the vehicle turning radius *R* is given by:

(19) $$\tan\Delta \theta \approx \frac{e}{R}$$

Bringing Equation (18) into Equation (19), when the value of *l* is determined, the range of values of Δθ is:

(20) $$\Delta \theta \leq \arctan\frac{\sqrt{l^{2}+s^{2}}}{R_{min}}$$

where $R_{min}$ represents the minimum turning radius of the robot. Therefore, the value of Δθ needs to be determined according to the constraint range of Equation (20), while *l* needs to be selected as appropriate according to the road width. *s* depends on the lookahead distance which can be set as a hyperparameter in NRTEB.

## 4. Results and Discussion

In this section, firstly, the trajectory of our method is compared with the advanced motion planning method in a constrained environment. Then, a simulation environment containing narrow corridors is built in Gazebo (https://github.com/osrf/gazebo, accessed on 15 January 2010) to compare the motion performance, reversing performance, and real-time performance of each method. Finally, we evaluate the reversing and obstacle avoidance performance of the proposed method in the narrow-road scenario on a real car-like robot platform. The experimental results of the article are reproducible with the same robot structure and the same experimental parameters. However, note that it needs to be assumed that the localization, perception, and global path planning modules work properly during the experiment.

### 4.1. Simulation and Analysis

The method of this paper is compared with the original TEB and MPC applied to the car-like robot model. The simulation experiments were conducted on a laptop with an Intel Core i5-6300HQ CPU 2.30 GHz × 4. The influence of algorithm parameters on the trajectory is also discussed.

#### 4.1.1. Trajectory Motion Performance Comparison

**Figure 8:**
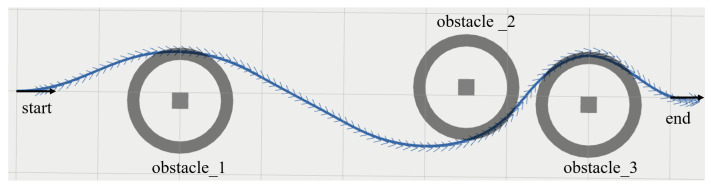
The trajectory generated by NRTEB. The curve is the motion trajectory and the arrows represent the heading angle of the planning state.

The motion planner usually updates the trajectory according to a certain frequency, so the trajectory has been dynamically varying as the robot moves and the environment changes. In order to accurately compare the motion performance of trajectories generated by different motion planners in the same environment, the start and end point are fixed to simulate the robot position and local target. Furthermore, three static obstacles are added in the direction of their motion. The posture of the planning start is ${\left(-4,0,0\right)}^{T}$. The posture of the planning end is ${\left(4,0,0\right)}^{T}$. All three obstacles are 0.2 m × 0.2 m × 0.2 m cubes, which are located at ${\left(3,-0.1\right)}^{T}$, ${\left(1.5,0.1\right)}^{T}$, and ${\left(-2,-0.1\right)}^{T}$, respectively. The planning parameters of the motion planners are shown in Table 1. The trajectory generated by NRTEB is shown in Figure 8.

**Figure 9:**
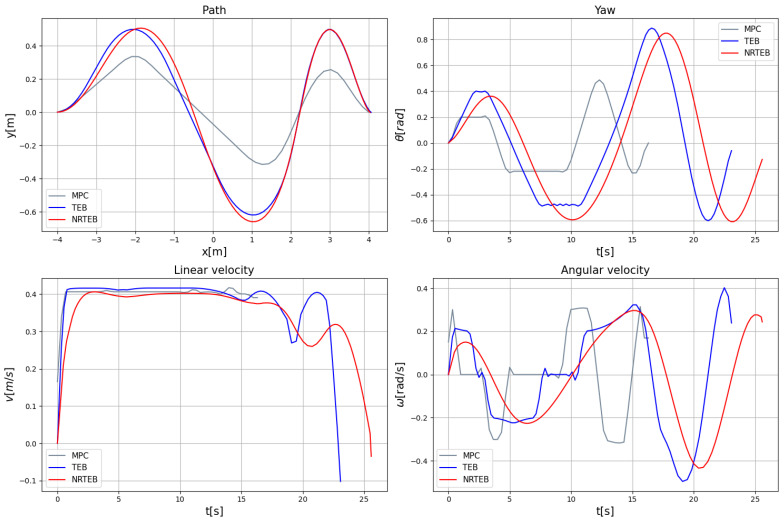
Comparison of MPC, TEB, and NRTEB in the path, heading angle, linear velocity, and angular velocity.

The motion performance of the trajectories generated by the three planners is shown in Figure 9. All three methods generate the correct obstacle avoidance path where MPC has the closest distance to the obstacle and the highest collision risk, but can reach the end point faster. While the paths generated by TEB and NRTEB are relatively safer. The speed profile of TEB and MPC is steeper and the yaw change is more rapid, which poses a more stringent challenge to the tracking stability. In contrast, the NRTEB has the smoothest speed profile, which significantly reduces jitter and oscillation during travel. This is important for logistics robots to avoid shaking and thus ensure the integrity of the products.

**Figure 10:**
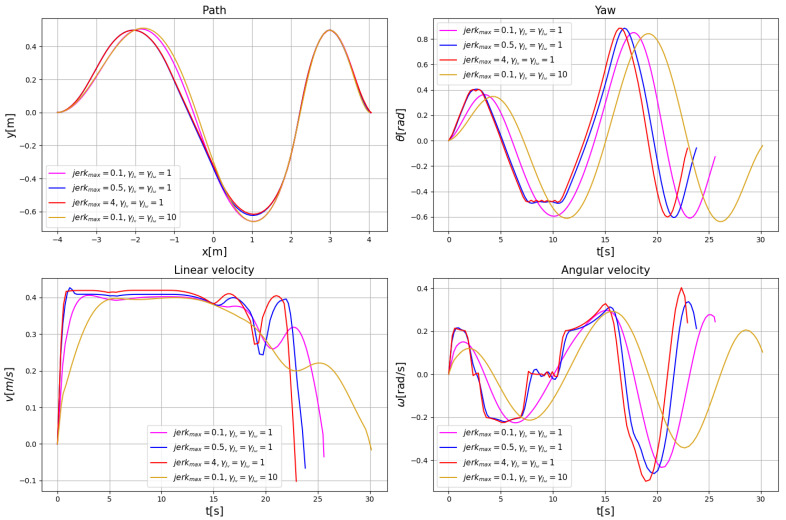
Motion curves with different planning parameters.

This paper also explores the effect of planning parameters on the performance of the NRTEB planner for trajectories. The different jerk bounds or optimization weights are set to compare the planning performance in the same environment, respectively. The results are shown in Figure 10. The tighter the jerk constraint, the greater the fluctuation of the planning path. It can be seen from the heading angle, linear velocity, and angular velocity curves that the tighter the jerk limit, the smoother the curve, and the longer the motion time at the same time, where the motion curve is smoothest for a jerk threshold of 0.1 and steepest for a jerk threshold of 4. An increase in the optimization weight of jerk further improves the motion smoothness and motion time. In this paper, we suggest that the constraint range of jerk is between 0.1 and 5, and the optimization weight of jerk is between 1 and 10. If there is a higher requirement for the motion time, the optimization weight of the trajectory motion duration can be further adjusted upward, thus making a balanced trade-off between motion smoothness and motion time.

#### 4.1.2. Tracking Performance Comparison

**Figure 11:**
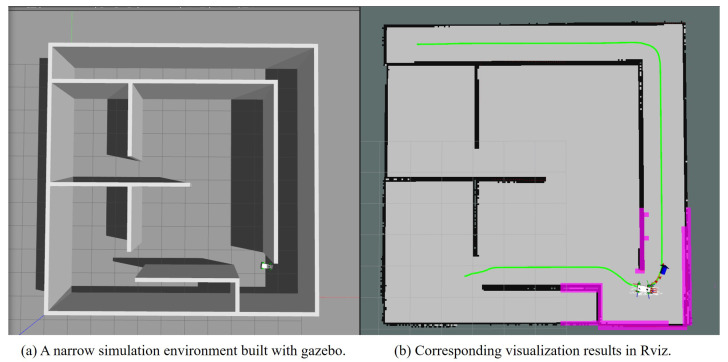
The simulation environment. The green curve represents the global path recorded in advance. The red curve with arrows represents the motion trajectory and the blue arrow indicates the local target.

Tracking the global path is an important goal of motion planning. The tracking error between the actual vehicle trajectory and the global path is an important indicator of planning performance when there is no obstacle interaction during navigation. A car-like robot model developed by MIT named racecar (https://github.com/mit-racecar/racecar, accessed on 28 February 2017) and a simulation environment built using Gazebo (https://github.com/osrf/gazebo, accessed on 15 January 2010) were used to conduct comparative experiments on tracking performance under Robot Operating System (ROS) (https://www.ros.org/, accessed on 23 May 2018). The vehicle is equipped with a single-line LIDAR on top to sense the surrounding environment obstacles. The environment size is 10 m × 10 m. The real-time motion state of the robot is feedback by the gazebo simulator. The global path is provided and recorded using the A* algorithm, and the same global path is kept published during the experiments. The simulation environment containing narrow corridors is shown in Figure 11. The key parameters for the simulation experiments are shown in Table 2. All parameters do not exceed the kino-dynamic constraints of the car-like robot model.

**Figure 12:**
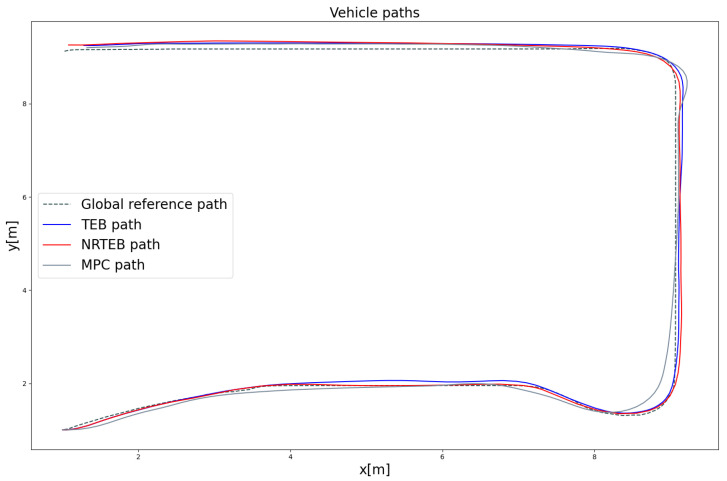
The traces of the robot’s odometry. The gray dashed line is the global reference path that was pre-recorded and published in the experiment. The starting pose is ${\left(1,1,0\right)}^{T}$ and the ending pose is ${\left(1,9,\pi \right)}^{T}$.

Firstly, the pre-recorded global path is published, and different motion planning algorithms are used to generate local motion trajectories. Then, the motion state of the robot is recorded in real-time from the starting point to the global target with a frequency of 2 Hz. The traces of the robot’s odometry are shown in Figure 12. The results of the actual robot path length and average lateral tracking error are shown in Table 3. Among them, MPC generates the shortest tracking path but has the largest tracking error, which makes it significantly riskier to collide with walls at the corners of narrow roads. TEB and NRTEB have a smaller tracking error, which allows the robot to stay in the middle of the narrow road. NRTEB requires a larger turning radius because it limits the jerk, but does not significantly affect the tracking performance.

The robot status data during navigation is shown in Table 4. It should be noted that, to reflect the degree of change in motion, the average values in Table 4 need to be averaged after taking the absolute values of the jerk data first. The motion of MPC took 117.495 s, the motion of TEB took 124.516 s, and the motion of NRTEB took 137.017 s. The results again verified that NRTEB can improve the speed smoothness by constraining the jerk of the trajectory. However, the side effect is to increase the steering radius and motion duration. It can be solved by adjusting the constraint range of jerk, the magnitude of jerk optimization weight and the weight of the trajectory motion duration, as described in the previous section. The average planning time consumption of MPC is 154 ms, which cannot guarantee real-time planning when the CPU computing power is limited, while the planning efficiency of TEB and NRTEB is significantly improved. It is further demonstrated that adding the jerk to the objective function for optimization does not significantly increase the planning time consumption since the jerk term is partial with respect to the whole trajectory, thus ensuring real-time planning.

#### 4.1.3. Comparison of Reversing Trajectory

**Figure 13:**
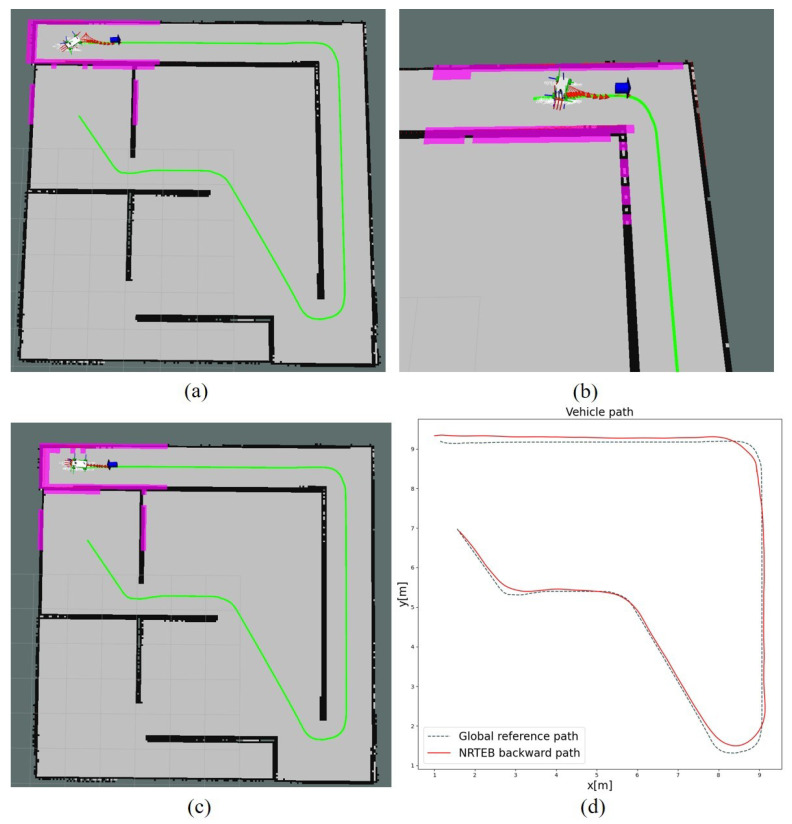
(**a**) Reversing trajectory generated by TEB. The red arrow represents the pose of the trajectory point and the blue arrow indicates the local target. (**b**) TEB’s reversing planning results in a narrow corner. (**c**) The straight reversing trajectory generated by NRTEB. (**d**) The comparison of the global path generated by A* algorithm and the reversing path with the NRTEB.

When the global path is behind the robot, TEB generates the parking trajectory for turning around, but this does not work on narrow roads, as shown in Figure 13a,b.

Due to the minimum steering radius constraint of the car-like robot, the trajectory is very likely to cause the robot to collide with the boundary or unable to turn around, thus leading to planning failure. MPC is even unable to generate a trajectory. NRTEB generates a straight reversing trajectory by modifying the reference heading of the local target, as shown in Figure 13c, which solves the problem of robot oscillation due to a minimum turning radius constraint when reversing on narrow roads. The initial state of the robot in this part of the experiment is the terminal state of the previous part, i.e., the robot pose is ${\left[1,9,\pi \right]}^{T}$ and the global target is set to ${\left[1.5,7,-\frac{\pi }{4}\right]}^{T}$. The global reference path generated by the A* algorithm located behind the robot is shown in Figure 13d. The diagram shows that NRTEB guides the robot out of the narrow dead zone and eventually reaches a new global target, which is not possible with the other two methods.

### 4.2. Realistic Obstacle Avoidance Scenarios

**Figure 14:**
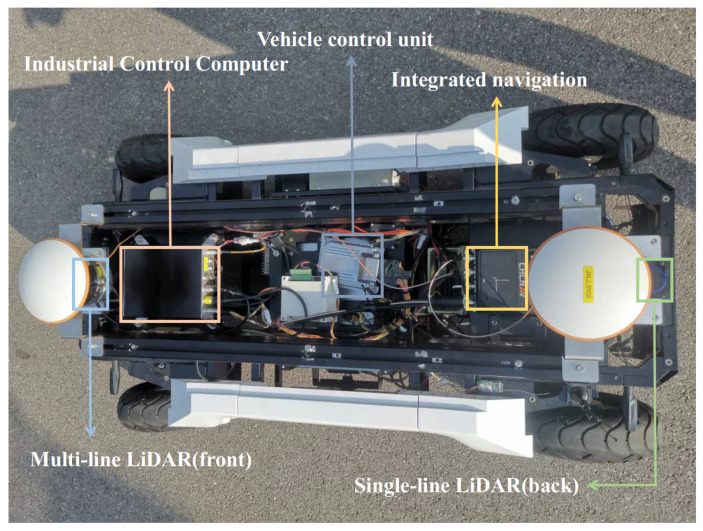
Self-developed autonomous ground vehicle.

Given that the simulation environment ignores the data transmission time delay and does not reflect the obstacle information of the robot in real narrow-road environments, the obstacle avoidance performance of NRTEB and TEB is compared through our self-developed car-like logistics robot platform, as shown in Figure 14. The components of the automated guided vehicle (AGV) are shown in Table 5. Vehicle chassis parameters are shown in Table 6.

**Figure 15:**
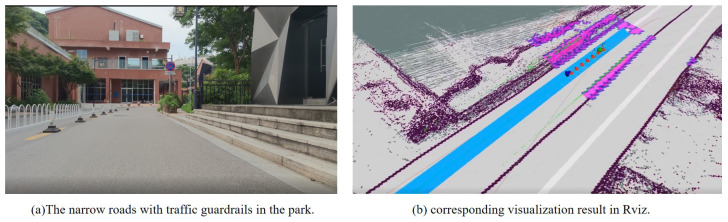
The real-world experiment. The blue line is the global reference path. The curve composed of red arrows is the result of the motion trajectory. The blue arrow represents the local target generated by NRTEB planner. The green sphere represents the tracking point selected in the local trajectory for the controller.

The AGV is tested on a 2.75 m-wide road with traffic guardrails. The planning parameters of NRTEB are shown in Table 7. When the vehicle is driving in the middle of the road, its distance from the edge of the road is 1.375 m, so set *l* to 1.0. According to Equation (20), the maximum value of Δθ is $67.59^{\circ }$, so choose Δθ as 60. Our system integrates with the global road network planning module in Autoware (https://www.autoware.org/, accessed on 1 August 2015) to provide a global reference path. Figure 15a shows the real environment captured by the camera, while Figure 15b shows the visualization of the AGV in Rviz.

**Figure 16:**
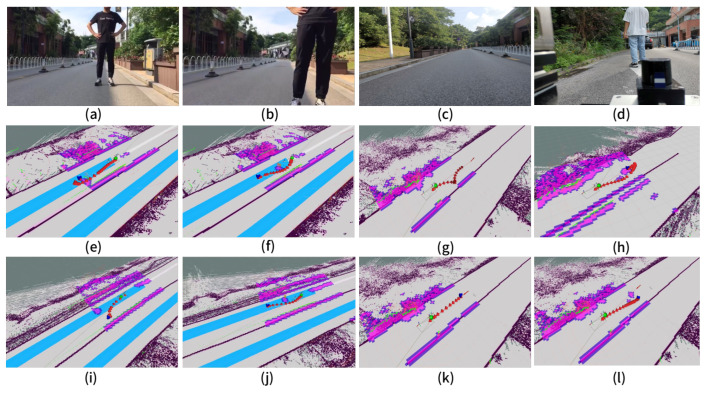
Real-world experimental results. (**a**–**d**) Are the pictures captured by the camera, (**a**,**b**) perform obstacle avoidance tests with pedestrians. (**c**,**d**) Are taken by the camera fixed on the back of the AGV. (**d**) Is used to test reverse obstacle avoidance. (**e**–**h**) Are the screenshots taken from Rviz when running TEB planner. (**i**–**l**) Are the screenshots when running the NRTEB planner.

To validate our planner, it is tested repeatedly on the loop road as shown in Figure 15. During navigation, pedestrians pass through the global reference path with irregular motion. A global target behind the robot is also set to test its reversal planning capabilities. The comparison experiments are conducted on the same narrow roads while keeping the same parameters. The results are shown in Figure 16.

When an obstacle appears on the global reference path of the AGV, as shown in Figure 16a, the TEB planner suffers from planning confusion which is caused by the fact that the search space becomes non-convex due to the intervention of dynamic obstacles, so that the TEB planning falls into local minima, as shown in Figure 16e, while NRTEB updates the optimization endpoint by sampling search to make the non-convex problem convex so it can avoid falling into local minima and generate optimal trajectories, as shown in Figure 16i. When the AGV has enough lateral displacement after following the first few points, the trajectory following the global reference path is generated again after the local target leaves obstacles at the next moment, as shown in Figure 16b,f,j, and at the current time, the TEB planner also generates the correct trajectory but closer to the obstacle, causing tracking to be more difficult. When the global target is behind the AGV and in the same direction as the AGV, the TEB planner generates a parking trajectory, which leads the vehicle to becoming trapped at the edge of the road by not being able to turn around on narrow roads, as shown in Figure 16g, and the NRTEB planner generates a reasonable reversing trajectory, as shown in Figure 16k. In addition, our improvement in obstacle avoidance is also applicable to the reversing scenario. If there are obstacles on the local target in the reversing process, as shown in Figure 16d, the TEB planner also does not generate a reasonable trajectory, as shown in Figure 16h, and the NRTEB planner looks for a new local target without obstacles as the endpoint of the current planning cycle to generate a correct trajectory, as shown in Figure 16l.

The planning performance of the real-world navigation is shown in Table 8. The results show that NRTEB significantly improves the smoothness of speed changes, although it increases some navigation elapsed time. When collision detection is performed, the planning time consumption is approximately 100 milliseconds and no more than 50 milliseconds during normal driving. If the computational power of IPC is insufficient, the size of the local cost map can be reduced appropriately and the maximum frequency of motion planning can be decreased at the same time. It should be noted that the NRTEB solution can be performed under the current parameters, but when the planning frequency and the local cost map update frequency are too high—causing the computational capacity of the IPC to be exceeded—the motion planning solution no longer converges, which can be solved by replacing the CPU with a more computationally powerful one.

## 5. Conclusions

In this paper, a motion planning algorithm for logistics car-like robots was proposed. It greatly improves the planning capability of a TEB algorithm in a narrow-road environment. It makes up for the shortcomings of TEB in terms of an unsmooth speed profile, unsafe reverse planning, and unstable dynamic obstacle avoidance. Compared with TEB, NRTEB plans a smoother speed profile. The linear jerk of the trajectory is 55.48% smaller and the angular jerk is 72.11% smaller than that of TEB. This improves the comfort of robot motion and greatly reduces the probability of good being damaged due to sudden speed changes. When navigating without obstacles, NRTEB tracks the global path in real time and stably. NRTEB’s tracking error is 24.46% smaller than MPC, and the planning time is reduced by 98.66%. In addition, NRTEB enables safe reverse navigation on narrow roads, which is not possible with the other two comparison methods. More significantly, real-world experiments demonstrate that NRTEB safely avoids dynamic obstacles in real time. It even achieves narrow-road obstacle avoidance whilst reversing.

NRTEB’s planning efficiency can be further improved. Future work will deploy local map update modules on GPU for parallel processing. We recommend applying this method to car-like robots that need to work frequently in narrow-road environments, including but not limited to logistics, inspection, search, and rescue, etc.

## Figures and Tables

**Table 1:** Kino-dynamic parameters and optimization parameters.

Constraint Parameters	Values	Application Scope
Maximum linear velocity (m/s)	0.4	MPC, TEB, NRTEB
Maximum angular velocity (m/s)	0.3	MPC, TEB, NRTEB
Maximum linear acc (m/s${}^{2}$)	0.5	MPC, TEB, NRTEB
Maximum angular acc (rad/s${}^{2}$)	0.5	MPC, TEB, NRTEB
Minimum distance to obstacle (m)	0.5	MPC, TEB, NRTEB
Maximum linear jerk (m/s${}^{3}$)	0.1	NRTEB
Maximum angular jerk (rad/s${}^{3}$)	0.1	NRTEB
The weight of linear velocity ($\gamma_{v}$)	1.0	TEB, NRTEB
The weight of angular vel ($\gamma_{\omega }$)	1.0	TEB, NRTEB
The weight of linear acc ($\gamma_{a_{v}}$)	1.0	TEB, NRTEB
The weight of angular acc ($\gamma_{a_{\omega }}$)	1.0	TEB, NRTEB
The weight of linear jerk ($\gamma_{J_{v}}$)	1.0	NRTEB
The weight of angular jerk ($\gamma_{J_{\omega }}$)	1.0	NRTEB
The weight of states	[2.0,2.0,2.0]	MPC
The weight of controls	[1.0,1.0]	MPC

**Table 2:** Simulation Parameters.

Constraint Parameters	Values
Maximum linear velocity (m/s)	0.5
Maximum angular velocity (m/s)	0.5
Maximum linear acc (m/s${}^{2}$)	1.0
Maximum angular acc (rad/s${}^{2}$)	1.0
Maximum linear jerk (m/s${}^{3}$)	2.0
Maximum angular jerk (rad/s${}^{3}$)	2.0
Local costmap size (m${}^{2}$)	5 × 5
Local costmap update frequency (Hz)	10.0
robot footprint size (m${}^{2}$)	0.4 × 0.2
Maximum steering angle (rad)	0.785
Motion planning frequency (Hz)	10.0

**Table 3:** Path length and average lateral tracking error.

	MPC	TEB	NRTEB
Path length (m)	23.186	23.507	23.644
Average lateral tracking error (m)	0.094	0.062	0.071

**Table 4:** Planning performance statistics.

		Min	Max	Average (abs)
MPC	$J_{v}$	−1.001	1.004	0.072
	$J_{\omega }$	−2.590	1.486	0.197
	*T*	0.041	488.954	154.117
TEB	$J_{v}$	−1.092	1.090	0.146
	$J_{\omega }$	−2.331	2.797	0.251
	*T*	0.045	15.508	1.488
NRTEB	$J_{v}$	−0.295	0.309	0.065
	$J_{\omega }$	−0.562	0.738	0.070
	*T*	0.050	18.920	2.069

$J_{v}$ (m/s^3^): The linear jerk. $J_{\omega }$ (rad/s^3^): The angular jerk. T (ms): Planning time consumption.

**Table 5:** Electronic components.

Components	Product Model
IPC	NVIDIA Jetson Xavier NX
GNSS	CHCNAV CGI410
LiDAR (front)	RoboSense-RS-LiDAR-16
LiDAR (back)	LEME-02A
Camera	Netcan 1080p
4G wireless	QUECTEL
Vehicle control unit	self-developed

**Table 6:** Vehicle chassis parameters.

Chassis Parameters	Values
Track (m)	0.60
Wheelbase (m)	0.98
Maximum steering angle (rad)	0.52
Minimum turning radius (m)	1.70
Rated load (kg)	200
Maximum velocity (m/s)	2.80
Maximum acceleration (no load) (m/s${}^{2}$)	2.5
Rated acceleration (m/s${}^{2}$)	0.77
Envelope size (m${}^{3}$)	1.30×0.70×0.46

**Table 7:** NRTEB parameters.

Planner Parameters	Values
$\gamma_{J_{v}}$	1.0
$\gamma_{J_{\omega }}$	1.0
*l* (m)	1.0
Δθ (${}^{\circ }$)	60
Lookahead_dist (m)	4.0
Maximum linear velocity (forward) (m/s)	2.50
Maximum linear velocity (backward) (m/s)	1.50
Maximum angular velocity (rad/s)	0.50
Maximum acceleration (m/s${}^{2}$)	0.75
Maximum linear jerk (m/s${}^{3}$)	1.0
Maximum angular jerk (rad/s${}^{3}$)	0.5
Minimum turning radius (m)	1.70
Minimum distance to obstacle (m)	0.5
Local costmap size (m${}^{2}$)	4×4
Local costmap update frequency (Hz)	10.0
Motion planning frequency (Hz)	10.0

$\gamma_{J_{v}}$: The weight of linear jerk. $\gamma_{J_{\omega }}$: The weight of angular jerk. *l*: The lateral obstacle avoidance distance. Δθ: The amount of angle change.

**Table 8:** Planning performance statistics.

		Min	Max	Average (abs)
TEB	$J_{v}$	−1.368	1.173	0.154
	$J_{\omega }$	−2.565	2.657	0.362
	*T*	10.854	117.142	50.627
	ΔT	—	—	217.514
NRTEB	$J_{v}$	−0.274	0.298	0.062
	$J_{\omega }$	−0.575	0.743	0.071
	*T*	11.947	120.620	53.613
	ΔT	—	—	239.265

$J_{v}$ (m/s^3^): The linear jerk. $J_{\omega }$ (rad/s^3^): The angular jerk. *T* (ms): Planning time consumption. ΔT (s): Time taken to run a complete navigation process.

## Appendix A

**Algorithm A1** processBackward
**Input:**$P_{robot}$: the robot current position; local_path: A local path intercepted from the global path; global_target: the global target **Output:**$\theta_{local\_target}$ 1: $D_{global}\leftarrow Euclidean\_distance\left(P_{global\_target},P_{robot}\right)$ 2: Set the reachable distance of the global target $\to D_{global\_near}$ 3: **if**$D_{global}>D_{global\_near}$**then** 4: Get the local_target(x,y,θ) from the last point of local_path→local_target 5: $D_{local}\leftarrow Euclidean\_distance\left(P_{local\_target},P_{robot}\right)$ 6: Calculate the direction vector between the robot and the local target $\to V_{\mathrm{lt}}$ 7: Calculate the direction vector of the robot $\to V_{\mathrm{robot}}$ 8: $dot\;product\leftarrow V_{\mathrm{lt}}\cdot V_{\mathrm{robot}}$ 9: **if** dotproduct<0 **then** 10: $\theta_{local\_target}\leftarrow \theta_{local\_target}+\pi$ 11: **else** 12: $\theta_{local\_target}\leftarrow \theta_{local\_target}$ 13: **end if** 14: **else** 15: $\theta_{local\_target}\leftarrow \theta_{global\_target}$ 16: **end if**

**Algorithm A2** processObstalcesOnLocalTarget
**Input:**$local\_target=(x_{rp},y_{rp})$, local_costmap, *l*, Δθ, Δl, Δs, Δϕ **Output:**new_local_target 1: Get the cost of local target from $local\_costmap\to C_{local}$ 2: **if**$C_{local}>0$**then** 3: $V_{\mathrm{dir}}=\left(v_{x},v_{y}\right)$ 4: $\theta \leftarrow \arctan\frac{v_{y}}{v_{x}}$ 5: $x_{gt}\leftarrow x_{lt}-l\sin\theta$ 6: $y_{gt}\leftarrow y_{lt}+l\cos\theta$ 7: $\theta_{gt}\leftarrow \theta +\Delta \theta$ 8: **if** $C_{gt}>0$ **then** 9: s←Euclidean_distance(robot,local_target) 10: seti=1 11: **while** Δθ−iΔϕ>0 and l−iΔl>0 and s−iΔs>0 **do** 12: $x_{as}\leftarrow x_{gt}+i\Delta l\cos\left(\frac{\pi }{2}-\theta \right)$ 13: $y_{as}\leftarrow y_{gt}-i\Delta l\sin\left(\frac{\pi }{2}-\theta \right)$ 14: $x_{sp}\leftarrow x_{as}-i\Delta s\cos\theta$ 15: $y_{sp}\leftarrow y_{as}-i\Delta s\sin\theta$ 16: $\theta_{sp}\leftarrow \theta +\Delta \theta -i\Delta \phi$ 17: **if** $C_{sp}==0$ **then** 18: **return** $new\_local\_target=(x_{sp},y_{sp},\theta_{sp})$ 19: **end if** 20: i=i+1 21: **end while** 22: **else** 23: **return** $guided\_turning\_point=(x_{gt},y_{gt},\theta_{gt})$ 24: **end if** 25: **else** 26: **return** $local\_target=(x_{lt},y_{lt},\theta_{lt})$ 27: **end if**
